# First brazilian consensus of advanced prostate cancer: recommendations for clinical practice

**DOI:** 10.1590/S1677-5538.IBJU.2016.0490

**Published:** 2017

**Authors:** Andre Deeke Sasse, Evanius Garcia Wiermann, Daniel Herchenhorn, Diogo Assed Bastos, Fabio A. Schutz, Fernando Cotait Maluf, George Coura, Igor Alexandre Protzner Morbeck, Juliano J. Cerci, Oren Smaletz, Volney Soares Lima, Ari Adamy, Franz Santos de Campos, Gustavo Franco Carvalhal, Leandro Casemiro Cezar, Marcos Francisco Dall´Oglio, Marcus Vinicius Sadi, Rodolfo Borges dos Reis, Lucas Nogueira

**Affiliations:** 1 Clínica Médica, Unicamp, Campinas, SP, Brasil;; 2 Sociedade Brasileira de Oncologia Clinica, Belo Horizonte, MG, Brasil;; 3 Instituto Nacional do Câncer, INCA, Rio de Janeiro, RJ, Brasil;; 4 Departamento de Oncologia, Instituto do Câncer do Estado de São Paulo, São Paulo, SP, Brasil;; 5 Departamento de Oncologia, Hospital Sirio-Libanes, São Paulo, SP, Brasil;; 6 Beneficiência Portuguesa de São Paulo, São Paulo, SP, Brasil;; 7 Departamento de Oncologia, Hospital Israelita Albert Einstein, São Paulo, SP, Brasil;; 8 Instituto do Câncer, Hospital das Clinicas da Faculdade de Medicina, Universidade de São Paulo, São Paulo, SP, Brasil;; 9 Universidade Católica de Brasilia, Taguatinga, DF, Brasil;; 10 Universidade de São Paulo, USP, São Paulo, SP, Brasil;; 11 Oncocentro, Belo Horizonte, MG, Brasil;; 12 Departamento de Urologia, Hospital Santa Casa, Curitiba, PR, Brasil;; 13 Departamento de Cirurgia e Departamento de Urologia, Pontifícia Universidade Católica do Rio Grande do Sul, Porto Alegre, RS, Brasil;; 14 Hospital Federal Cardoso Fontes, Rio de Janeiro, RJ, Brasil;; 15 Departamento de Urologia, Universidade de São Paulo, USP, São Paulo, SP, Brasil;; 16 Universidade Federal de São Paulo, São Paulo, SP, Brasil;; 17 Faculdade de Medicina de Ribeirão Preto, Universidade de São Paulo, Ribeirão Preto, SP, Brasil;; 18 Departamento de Urologia, Hospital das Clínicas, Universidade Federal de Minas Gerais, Belo Horizonte. MG, Brasil

**Keywords:** Prostatic Neoplasms, Practice Guideline [Publication Type], Diagnosis

## Abstract

**Introduction:**

Prostate cancer still represents a major cause of morbidity, and still about 20% of men with the disease are diagnosed or will progress to the advanced stage without the possibility of curative treatment. Despite the recent advances in scientific and technological knowledge and the availability of new therapies, there is still considerable heterogeneity in the therapeutic approaches for metastatic prostate cancer.

**Objectives:**

This article presents a summary of the I Brazilian Consensus on Advanced Prostate Cancer, conducted by the Brazilian Society of Urology and Brazilian Society of Clinical Oncology.

**Materials and Methods:**

Experts were selected by the medical societies involved. Forty issues regarding controversial issues in advanced disease were previously elaborated. The panel met for consensus, with a threshold established for 2/3 of the participants.

**Results and Conclusions:**

The treatment of advanced prostate cancer is complex, due to the existence of a large number of therapies, with different response profiles and toxicities. The panel addressed recommendations on preferred choice of therapies, indicators that would justify their change, and indicated some strategies for better sequencing of treatment in order to maximize the potential for disease control with the available therapeutic arsenal. The lack of consensus on some topics clearly indicates the absence of strong evidence for some decisions.

## INTRODUCTION

Except for skin cancer, prostate cancer is the most prevalent malignant neoplasm in men around the World. In Brazil, the number of estimated new patients diagnosed with prostate cancer in 2016 was 61,200, with an estimated risk of 61.82 new patients for every 100,000 men. Every year 13,000 deaths due to prostate cancer are estimated in Brazil (1).

Although associated with a pattern of indolent disease, mainly when diagnosed during population screening, metastatic prostate cancer is an important public health issue, with high mortality rate and complex treatment. Even when initial androgen deprivation is efficient, the disease may inevitably progress to a resistant form. In that case, disease-related death is high. Also, quality of life of patients with prostate cancer resistant to castration (PCRC) is frequently altered due to symptoms associated to fatigue, bone metastasis pain, etc.

After a long period of limited therapeutic options (secondary hormonal treatments), over the last years several clinical studies introduced new treatments that increased free-progression survival, global survival and quality of life (2-7). The better understanding of the role of androgenic pathway (including intracellular synthesis of androgens and the role of androgenic receptors (AR) in prostate cancer, and the knowledge that disease progression remains associated to the activation of that pathway) was fundamental for the development of new drugs that are able to improve clinical outcomes. Due to that fact, the term “prostate cancer refractory to hormonal therapy” was replaced by a more adequate – prostate cancer resistant to castration (PCRC). Also, nowadays there are two available active cytotoxic drugs that may have a positive impact on survival of patients, that would not have had benefits with traditional chemotherapy (6,8).

In spite of the discussed recent advances, there is still great heterogeneity of therapeutic approaches for metastatic prostate cancer. In Brazil, it must be stressed the delayed inclusion of new technologies and regional differences related to access to new drugs and specialists. It is important to evaluate the possibility to include new therapeutic developments in public and private health services in Brazil. With that in mind, a panel of Brazilian specialists discussed and proposed a document with therapeutic recommendations for the treatment of advanced prostate cancer.

### Objective

The present consensus is a co-joint initiative of the Brazilian Society of Clinical Oncology (BSCO) and Brazilian Society of Urology (SBU). The objective is to provide guidelines to help clinical decisions by physicians that treat patients with advanced prostate cancer (mainly urologists, clinical oncologists and radio-oncologists).

### Methodology

Experts were indicated by BSU and BSCO. Apart from the moderator,18 leading professionals in the field, from different regions of Brazil, were selected. The panel was composed by 8 clinical oncologists, 8 urologists and 2 nuclear medicine physicians.

The consensus format was adapted by the model of the *St. Gallen Advanced Prostate Cancer Consensus Conference* (APCCC) 2015 (9).The items of that consensus were previously turned into clinically relevant questions and posteriorly distributed to participants, in order to systematic review and critically analyze the information. Questions focused on treatment and follow up of patients with metastatic prostate cancer sensible or resistant to castration. Epidemiologic data, treatment of localized disease or screening were not addressed by the consensus. Initially, 60 questions were distributed to a subgroup of 8 selected specialists. A preliminary meeting took place to select the relevant questions, to discuss the best format of questions and to validate the final draft. Also, it was defined which specialists would be responsible for writing the answers to each of the questions. Next, 40 selected questions were formulated to participants, who had a 2-month period of time to critically analyze the studies on the theme, according to pre-defined levels of evidences (10) (See Appendix).

The panel of the Brazilian Consensus of Advanced Prostate Cancer took place in November 4th, 2015, at Rio de Janeiro, Brazil. It was used the Delphi modified method to obtain the consensus (11). Participants agreed to stablish a consensus limit of 2/3 of participants. To each question, it was presented the existing options followed by panelists vote. In case the participant thought that he/she did not have enough experience to vote, or felt unable to pick up an answer, or presented conflicting interests, it was chosen the option “does not apply to my practice/I rather not vote”. Under the guidance of the moderator, the panel debated all conflicting data, or when there was no consensus, it was proposed a second vote. In case of maintenance of lack of consensus, it was made clear in the manuscript the lack of agreement on the subject. It was opted to expose in the manuscript all questions and answers, with the votes and respective percentages. The manuscript was written based on the records and meeting minutes, being subsequently approved by all participants.

## RESULTS

### Development of the consensus and panel discussion

The results of voting with or without consensus are available in the Addendum. Next, we present the main conclusions of the Brazilian Consensus of Advanced Prostate Cancer:

### Initial hormonal treatment of advanced prostate cancer sensitive to castration

The development and progression of prostate cancer is highly influenced by the androgenic pathway. The main objective of hormonal treatment is to lower androgen action in the organism, avoiding cellular multiplication through signaling pathways present in sensitive cells. GnRH analogues (monotherapy) are the most used drugs with that objective as first line of treatment. However, the existence of other valid modalities of treatment, such as the use of Gn-Rh antagonists, sub-capsular orchiectomy or even the association of testosterone suppression and peripheral anti-androgens were reminded by the panel.

**Figure 1:**
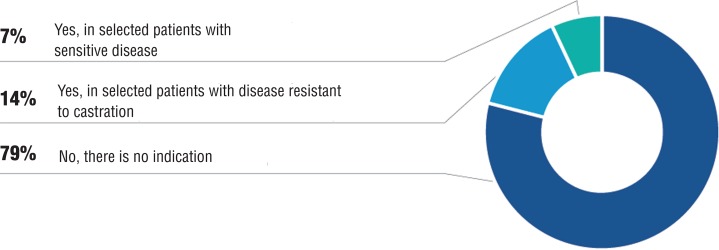
Is there any indication for the use of ciproterone in the treatment of metastatic prostate cancer?

The use of ciproterone acetate was not indicated by the panel, with 79% of concordance (Figure-1). Although still widely used, literature data show worsening of survival of patients taking ciproterone, alone or combined to androgen suppression (12).

There was consensus on the recommendation of serum concentration of testosterone below 50 ng/dL for the definition of castration. However, it was stressed the fact that literature shows a potential clinical benefit to maintain patients with lower levels (such as 20 ng/dL).

**Figure 2:**
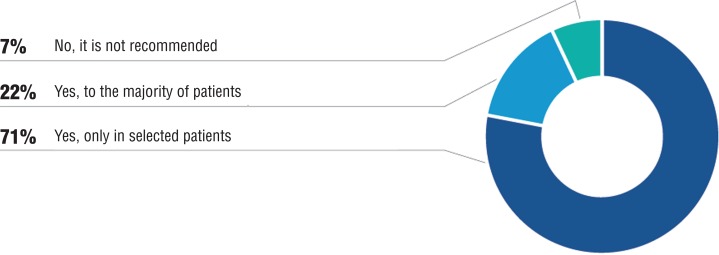
Is it recommended to use intermittent androgen deprivation instead of continous androgen suppression in patients with radiologically documented metastasis that reach adequate PSA lowering?

Although with benefits on survival, some studies suggest that testosterone suppression in the long follow-up has been associated to important side effects, along with worsening of quality of life (13). Patients with well controlled disease may have some benefit with temporary withdrawal of hormonal blockage, adopting an intermittent treatment regimen. However, randomized studies are controversial in relation to efficacy and safety of intermittent hormonal blockage. Based on recent data, 71% of panelists agreed that intermittent hormonal blockage may be recommended to asymptomatic patients, with radiologically confirmed metastasis and with correct lowering of PSA levels (usually above 90% and PSA < 4 ng/mL) (Figure-2).

### Chemotherapy of prostate cancer sensitive to castration

Two prospective and randomized trials support the inclusion of docetaxel chemotherapy in the initial treatment using androgen suppression in patients with metastatic prostate cancer in a phase still sensitive to castration: CHAARTED (14) and STAMPEDE (15).

However, a randomized study failed to demonstrate the benefit of early chemotherapy, GETUG-AFU 15 (16). In the CHAARTED study, the inclusion of docetaxel in the group of patients with low volume disease did not demonstrate increase of median global survival. However, it is important to remind that in this group the number of patients was reduced, and the statistical analysis was inadequate due to the low number of deaths observed in that group. Based on the existent studies, 73% of the members of the panel agreed that for patients with metastatic prostatic cancer, with high volume disease, it is recommended the use of docetaxel associated to androgenic suppression. The panel considered the criteria adopted by the CHAARTED study the most adequate to consider the definition of high volume disease: presence of visceral disease and/or four or more bone metastasis, at least one outside the pelvic ring and vertebral column.

### Local therapy in patients with oligo-metastatic disease

Local treatment of oligo-metastatic or metastatic disease, using radiotherapy, cryotherapy, HIFU or radical prostatectomy, in addition to systemic treatment did not show any benefit in relation to single systemic treatment in controlled prospective or randomized studies, with good levels of evidence; however, recent studies have demonstrated benefit on survival and quality of life in a subgroup of patients with minimum metastatic disease submitted to radical prostatectomy or radiotherapy. However, there was consensus on the fact that, at the moment, it is not recommended local treatment of the primary tumor in patients newly diagnosed with oligo-metastatic disease.

### Prostate cancer resistant to castration - MO

The panel agreed unanimously that confirmed PSA progression and/or radiologic progression of patients under androgenic suppression define disease resistant to castration, while patients present serum testosterone in castration levels. In the absence of detectable metastasis by image exams, there was a 69% consensus of panelists that there is no indication of additional treatment, due to absence of studies that have shown any relevant benefit.

**Figure 3:**
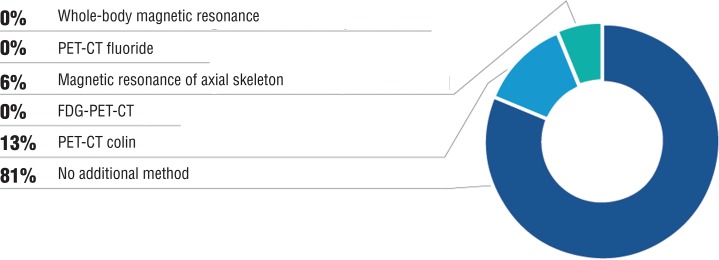
In patients with biochemical progression and negative scintigraphy and computorized tomography, which other diagnostic method should be used?

There was also 81% of agreement that in patients with biochemical progression and negative computerized scan of thorax/abdomen + bone scintigraphy, no other diagnostic method is indicated (Figure-3).

In asymptomatic patients, 69% of panelists agreed to realize periodically image exams in order to screen for metastasis, and not to wait the appearance of symptoms. However, it is not possible to stablish the periodicity of those exams.

### Prostate cancer resistant to castration with metastasis (PCRCm)

There is a significant heterogeneity of prostate cancer among individuals and also in the same patient. In spite of that, there are no reliable biomarkers at present to define an individual therapy. In most occasions a new biopsy of metastatic lesion for histological exam does not alter the therapeutic decision and there are no studiesthat have shown any efficacy with this strategy. With that in mind, 72% of panelists agreed that it is not necessary to perform routine biopsy at the site of metastasis for patients with progressing PCRCm and with accessible lesions. An exception may be made for patients with suspicion of evolution to neuro-endocrine tumor lines, such as those with very low PSA and development of visceral metastasis.

For control of progression and therapy response, 94% of panelists agreed on re-staging of patients with PCRCm before starting a new line of treatment. PSA progression only, without clinical worsening or radiological progression, does not justify change of treatment, what is supported by 88% of panelists.

All members of the panel agreed on the control using PSA for treatment response to new hormonal agents, associated to regular image exams and clinical evaluation (including analysis of patients symptoms).

In relation to the best initial treatment of PCRCm, there are many aspects regarding the different profiles of patients that potentially are candidate to chemotherapy or the use of new hormonal agents. Gleason score is not recommended as parameter for evaluation of choice between chemotherapy or abiraterone/enzulatamide, according to 88% of panelists. Also, the duration of the response to androgen suppression, the presence of visceral metastasis and the presence or not of symptoms were not considered adequate criteria for the choice between chemotherapy or abiraterone/enzalutamide.

In asymptomatic or slightly symptomatic patients, the panel remained divided without consensus when asked if they would recommend the use of docetaxel as first line of treatment in cases where abiraterone/enzalutamide was available. However, when asked if they would recommend the use of abiraterone/enzalutamide in that same population, the consensus reached 88% to indicate the drugs if all options were available, including the drug docetaxel.

In symptomatic patients, there was consensus of 88% of panelists that, if available, it would be recommended the use of abiraterone/enzalutamide as first line of treatment for that population of patients associated to androgenic suppression. In addition, there was an 88% agreement forthe eventual recommendation of the use of chemotherapy with docetaxel as first line of treatment for patients with symptomatic PCRCm associated to androgenic suppression with GnRH analogues.

For new agents that act on the hormonal axis, there was 94% of agreement of panelists that the order of use (initially abiraterone or enzalutamide) doesn’t matter in the treatment of patients with PCRCm.

**Figure 4:**
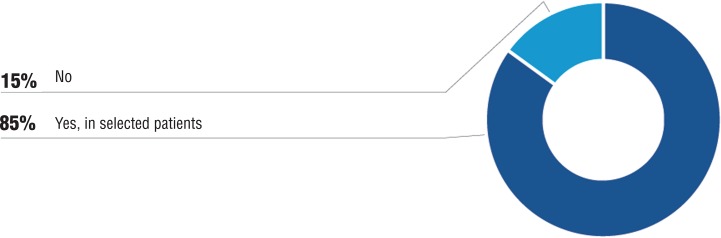
In an environment without any restrict access to other therapeutic options, is there indication of re-treatment with docetaxel?

Initially, the panel was divided but after discussion reached 85% of agreement with the possibility of re-treatment with docetaxel of selected patients, even in scenarios without restrict access to other options of treatments. Patients with good initial response to docetaxel, with good tolerability and delayed progression, would be better candidates to re-treatment (Figure-4).

### Bone therapy in metastatic prostate cancer

Zoledronic acid and more recently denosumabe are usually used to reduce the risk of bone fractures in patients with PCRCm. Although denosumabe had shown superior reduction of severe bone accidents in relation to zolendronate, it was not observed increase of survival (17); both medications are adequate and may be used. The panel agreed 100% in recommend the use of osteolysis inhibitors in patients with PCRCm and bone metastasis. And also agreed 100%to not recommend the use of osteolysis inhibitors to prevent bone fractures in patients with disease sensitive to castration with bone metastasis.

Ninety-three percent of panelists agreed that Radium-223 may be used, when available, in patients with PCRCm and symptomatic bone metastasis without visceral metastasis, in patients already treated with docetaxel or not. In asymptomatic patients, the panel does not recommend (94%) the use of Radium-223. In exceptional situations, it may be used radio-pharmaceutics such as those that emit beta particles (samarium/strontium) when the objective is to lower bone pain. These therapies are associated to pain improvement in 40-95% of patients (18). A consensus of 82% was reached that samarium and strontium may also be used as palliatives to treat bone pain in selected patients.

### Sequencing of therapies in metastatic prostate cancer resistant to castration (PCRCm)

According to randomized studies using abiraterone (COU-AA-301 and COU-AA-302) (4, 5) or enzalutamide (AFFIRM and PREVAIL) (2, 3), 10 to 30% of patients show progression of radiological or clinical disease at first evaluation of response. These patients are considered primarily refractory to these new hormonal agents and should be spared of an inadequate treatment if there was any biomarker that could predict response. AR-V7 is an androgen-receptor variant that lost the site of attachment to androgen and remains active regardless stimulation by androgens. In consequence, the new hormonal agents abiraterone and enzalutamide would fail to control those patients’ disease (19). However, the methodology to detect AR-V7 in circulating tumor cells is very difficult and not commercially available. Due to the difficulty of methodology to detect this kind of biomarker described in the studies, 93% of panelists agreed that currently there is no indication for the use of biomarkers like AR-V7 for decision between abiraterone/enzalutamide or chemotherapy.

In relation to sequencing of treatments, it was agreed that the time to response to docetaxel should not be considered when choosing subsequent treatments. The panel also recommended the use of cabazitaxel for patients with PCRCm after sequence of treatment with abiraterone/enzalutaide and docetaxel.

Unanimously, the panel considered that abiraterone, enzalutamide, cabazitaxel and Radium-223 may be used in patients with response to docetaxel and with progression of disease in less than three months following suspension of docetaxel.

## CONCLUSIONS

This consensus was proposed to provide valuable information for treatment guidance and use of current knowledge of scientific literature in Brazilian reality.

The treatment of patients with advanced prostate cancer is complex, due the existence of several different therapies, with different response and toxicity profiles. It should be pointed out that not always there are enough evidence to compare them. The choice of therapies may be individualized depending on specific clinical characteristics of each patient and some may be preferable.

The panel indicated recommendations regarding preferential choice of therapies, guidelines to justify their change and some strategies for sequencing of treatments, in order to maximize the control of the disease with the available drugs.

The lack of consensus in some topics clearly indicates the lack of strong evidences for some decision making.

In the proposal of recommendations, it was considered the potential benefits, the availability of drugs in Brazil, the costs and the side effects and involved risks.

These guidelines must be regarded as orientations. It is important to have in mind that the use of these recommendations does not warrant an adequate clinical disclosure for all patients. Final judgement on which clinical procedure or treatment plan of a specific patient must be made by the physician according to discussion of options with the patient, to the diagnosis and available therapeutic options. However, it is recommended that significant different approaches during clinical practice in relation to these guidelines must be justified and their reason correctly documented.

## References

[1] (2016). Estimativas 2016 - Incidência de Câncer no Brasil.

[2] Beer TM, Armstrong AJ, Rathkopf DE, Loriot Y, Sternberg CN, Higano CS (2014). Enzalutamide in metastatic prostate cancer before chemotherapy. N Engl J Med.

[3] Scher HI, Fizazi K, Saad F, Taplin ME, Sternberg CN, Miller K (2012). Increased survival with enzalutamide in prostate cancer after chemotherapy. N Engl J Med.

[4] Ryan CJ, Smith MR, Fizazi K, Saad F, Mulders PF, Sternberg CN (2015). Abiraterone acetate plus prednisone versus placebo plus prednisone in chemotherapy-naive men with metastatic castration-resistant prostate cancer (COU-AA-302): final overall survival analysis of a randomised, double-blind, placebo-controlled phase 3 study. Lancet Oncol.

[5] Fizazi K, Scher HI, Molina A, Logothetis CJ, Chi KN, Jones RJ (2012). Abiraterone acetate for treatment of metastatic castration-resistant prostate cancer: final overall survival analysis of the COU-AA-301 randomised, double-blind, placebo-controlled phase 3 study. Lancet Oncol.

[6] Bono JS de, Oudard S, Ozguroglu M, Hansen S, Machiels JP, Kocak I (2010). Prednisone plus cabazitaxel or mitoxantrone for metastatic castration-resistant prostate cancer progressing after docetaxel treatment: a randomised open-label trial. Lancet.

[7] Parker C, Nilsson S, Heinrich D, Helle SI, O’Sullivan JM, Fosså SD (2013). Alpha emitter radium-223 and survival in metastatic prostate cancer. N Engl J Med.

[8] Tannock IF, Wit R de, Berry WR, Horti J, Pluzanska A, Chi KN (2004). Docetaxel plus prednisone or mitoxantrone plus prednisone for advanced prostate cancer. N Engl J Med.

[9] Gillessen S, Omlin A, Attard G, Bono JS de, Efstathiou E, Fizazi K (2015). Management of patients with advanced prostate cancer: recommendations of the St Gallen Advanced Prostate Cancer Consensus Conference (APCCC) 2015. Ann Oncol.

[10] (2011). Levels of Evidence.

[11] Milholland AV, Wheeler SG, Heieck JJ (1973). Medical assessment by a Delphi group opinion technic. N Engl J Med.

[12] Prostate Cancer Trialists’ Collaborative Group (2000). Maximum androgen blockade in advanced prostate cancer: an overview of the randomised trials. Lancet.

[13] Carneiro A, Sasse AD, Wagner AA, Peixoto G, Kataguiri A, Neto AS (2015). Cardiovascular events associated with androgen deprivation therapy in patients with prostate cancer: a systematic review and meta-analysis. World J Urol.

[14] Sweeney CJ, Chen YH, Carducci M, Liu G, Jarrard DF, Eisenberger M (2015). Chemohormonal Therapy in Metastatic Hormone-Sensitive Prostate Cancer. N Engl J Med.

[15] James ND, Sydes MR, Clarke NW, Mason MD, Dearnaley DP, Spears MR (2016). Addition of docetaxel, zoledronic acid, or both to first-line long-term hormone therapy in prostate cancer (STAMPEDE): survival results from an adaptive, multiarm, multistage, platform randomised controlled trial. Lancet.

[16] Gravis G, Fizazi K, Joly F, Oudard S, Priou F, Esterni B (2013). Androgen-deprivation therapy alone or with docetaxel in non-castrate metastatic prostate câncer (GETUG-AFU 15): a randomised, open-label, phase 3 trial. Lancet Oncol.

[17] Fizazi K, Carducci M, Smith M, Damião R, Brown J, Karsh L (2011). Denosumab versus zoledronic acid for treatment of bone metastases in men with castration-resistant prostate cancer: a randomised, double-blind study. Lancet.

[18] Finlay IG, Mason MD, Shelley M (2005). Radioisotopes for the palliation of metastatic bone cancer: a systematic review. Lancet Oncol.

[19] Antonarakis ES, Lu C, Wang H, Luber B, Nakazawa M, Roeser JC (2014). AR-V7 and resistance to enzalutamide and abiraterone in prostate cancer. N Engl J Med.

